# The indispensable intermediaries: a qualitative study of informal caregivers’ struggle to achieve influence at and after hospital discharge

**DOI:** 10.1186/1472-6963-14-331

**Published:** 2014-07-30

**Authors:** Line Kildal Bragstad, Marit Kirkevold, Christina Foss

**Affiliations:** 1 Department of Nursing Science, Institute of Health and Society, University of Oslo, P.O. Box 1130, Blindern, NO-0318 Oslo, Norway

**Keywords:** Informal caregivers, Family, Consumer participation, Aged 80+, Informal help, Formal help, Home health care services

## Abstract

**Background:**

The care policy and organization of the care sector is shifting to accommodate projected demographic changes and to ensure a sustainable model of health care provision in the future. Adult children and spouses are often the first to assume care giving responsibilities for older adults when declining function results in increased care needs. By introducing policies tailored to enabling family members to combine gainful employment with providing care for older relatives, the sustainability of the future care for older individuals in Norway is more explicitly placed on the family and informal caregivers than previously. Care recipients and informal caregivers are expected to take an active consumer role and participate in the care decision-making process. This paper aims to describe the informal caregivers’ experiences of influencing decision-making at and after hospital discharge for home-bound older relatives.

**Methods:**

This paper reports findings from a follow-up study with an exploratory qualitative design. Qualitative telephone interviews were conducted with 19 informal caregivers of older individuals discharged from hospital in Norway. An inductive thematic content analysis was undertaken.

**Results:**

Informal caregivers take on comprehensive all-consuming roles as intermediaries between the care recipient and the health care services. In essence, the informal caregivers take the role of the active participant on behalf of their older relative. They describe extensive efforts struggling to establish dialogues with the “gatekeepers” of the health care services. Achieving the goal of the best possible care for the care recipient seem to depend on the informal caregivers having the resources to choose appropriate strategies for gaining influence over decisions.

**Conclusions:**

The care recipients’ extensive frailty and increasing dependence on their families coupled with the complexity of health care services contribute to the perception of the informal caregivers’ indispensable role as intermediaries. These findings accentuate the need to further discuss how frail older individuals and their informal caregivers can be supported and enabled to participate in decision-making regarding care arrangements that meet the care recipient’s needs.

## Background

Population projections show a significant increase of the older population in the European countries over the next 40 years [1]. Although the increase is not as dramatic in Norway [2], which is the setting of this study, as in some of the other European countries [1,3], the old age dependency ratio is cause for concern with regards to accommodating the increasing need for health care services in the aging population [1,3].

During the last 20 years we have seen a substantial change in primary care policy resulting in a retrenchment of institutional care in the municipalities in Norway and other European countries [4-6]. To compensate for this downscaling of care institutions, there has been an expansion of the municipal home-care services [1,7,8]. These home-care service developments coincide with the increased policy emphasis on aging in place seen in Norway and throughout the Western world [1,9].

The care policy and organization of the care sector is shifting to accommodate projected demographic changes [1,8] and to ensure a sustainable model of health care provision in the future [3,10,11]. When welfare states are under pressure and are obliged to discuss potential prioritizing and rationing of welfare services, the growing interest in informal care is noticeable [8,12].

Contemporary policy documents acknowledge that in order to maintain the level of support provided by informal caregivers today, a new “modern policy for informal care” that looks closely at the relationship between employment and caregiving in a more future-oriented manner is required [3,10,11]. By introducing policies tailored to enabling family members to combine gainful employment with providing care for older relatives, the sustainability of the future care for older individuals in Norway is more explicitly placed on the family and informal caregivers than previously [3,10,11].

### Formal health care services

The premise that health care is a public responsibility has traditionally been a core element of the Nordic welfare state [13]. This welfare state model differs from other models in that the arrangements between the state, market, and family strongly favor placing the responsibility with the welfare state [13]. This means that the state is established as the preferred and dominant provider of care, a model that is collectively supported by the Norwegian population [14]. The public services in Norway are based on the principle of universalism, which involves a uniform standard of services across all municipalities and counties in a model that incorporates all citizens in one universal system [13]. A central tenet of the Nordic welfare state model is to ensure provision of health care services and institutional care according to the citizens’ needs, independent of personal wealth, availability of family members to deliver informal care, or place of residence [13,15]. Nevertheless, the substantial welfare state expansion in the post-war era has not eroded filial obligations in Norway [14]. Despite placing the primary responsibility with the formal health care services, the adherence to filial obligation norms is expressed in a resilient belief that the family has a responsibility to support their older relatives [13,14,16]. This belief is demonstrated through the consistently high levels of care provided by informal caregivers of home-bound older relatives over the past 20 to 30 years [3,16,17], although it is significantly higher in countries with less developed formal home care services [1].

In Norway, the formal health care services are primarily public services organized in a two-tier model that consists of the specialist health care services at one tier and primary health care services at the other tier. The hospitals are a part of the specialist health care services. Hospitals are owned and financed by the Ministry of Health and Care Services and managed by regional health enterprises. Long-term care is part of the primary health care services, which are owned, financed, and managed by local municipalities.

### Informal care

In the Nordic countries, research on informal care has received less attention compared to the amount of research on formal care [16,18]. However, this trend changed during the 1990s [16,18]. In the international research community, research on informal care has been concerned with who provides informal care [19,20] and what kind of help and support informal caregivers provide [21-23]. Another perspective has been on what motivates family members to provide informal care to older relatives [24-27]. It is widely recognized that informal caregiving can be challenging on several different levels, thus, a significant amount of research concerns the caregiver burden of informal caregivers [28-31].

Research has shown that, traditionally, spouses, adult children, and extended family members are the first to assume caregiving responsibilities for older relatives when care needs arise [32]. The family assumes an important role in providing practical assistance and providing essential emotional support during hospitalization and after discharge [17,33]. In addition, informal caregivers play an important role in supporting their older relative in health care consultations [23,34], managing information [35], and in negotiating formal care in the community [36,37] by ensuring high-quality services when patients are not able to demand this for themselves [38]. However, changing family structures and increased mobility in and across country borders [8] pose challenges to the availability of informal care for older individuals living in the community.

### Consumer participation

The concept of patients as consumers has gained momentum in the health care services during recent decades [39]. Consumer participation has become a way to make the health care services responsive to individual needs and preferences by giving decision rights to those who receive medical care [40]. This shift has challenged the paternalistic model that traditionally dominated the relationship between patients and health care services, in which the patient is a passive recipient of care, while the health care personnel make decisions based on their expert medical knowledge [41]. This shift toward increased patient autonomy entails redefining the patient role from passive recipient to active participant [41]. The concept of increased autonomy and consumer participation has become an established ideal in the health care legislation, providing patients and his or her family a legal right to participate in the decision-making process to influence the choice of available treatment options and how treatment and care is provided [42]. Care recipients and their informal caregivers are encouraged to use their consumer influence to request high-quality services and are able to lodge complaints when services are not satisfactory [41]. However, this may not always work in practice, because older patients in particular may find it difficult to act as consumers, and they often practice participation in a subtle and discrete way [43]. Thus, older individuals come to depend on others, mainly their family, to represent them when the quality of care is not satisfactory [38].

### Informal caregiver participation in the discharge process

Informal caregivers’ involvement in the discharge process is found to increase their satisfaction with discharge planning, continuity of care, feelings of preparedness, and acceptance of the caring role and to increase the well-being of patients and their informal caregivers [44,45]. Involving family members has also been shown to improve the care recipient’s participation in the decision-making process [46,47]. Moreover, it is recognized that informal caregivers’ satisfaction with the discharge process influences the patients’ satisfaction and even influences the patient outcome positively [45]. However, research indicates that informal caregivers’ involvement in discharge planning is limited [48]. Family members are rarely consulted despite their potential as important resources in the discharge process and not least as important sources of support for the patients in the first post-discharge period [49,50].

Research on the transition between the home and hospital has emphasized the importance of collaboration between relatives of older patients and formal caregivers, indicating the need for a new, more active role for relatives as partners in decision-making at admission and discharge [51]. In the hospital setting, informal caregivers struggle to be more involved [52]; however, participation can be hampered by a lack of dialogue between formal and informal caregivers [52,53]. Furthermore, research has shown that informal caregivers can act as a “bridge” between the patient and formal care, facilitating formal care [54] by initiating the process of acquiring formal help for their home-bound older relatives [37].

### Rationale of the study

The contemporary demographic changes put pressure on formal and informal care delivery in the municipalities after hospital discharge. Consumer participation in discharge planning is encouraged to ensure continuity of care and care delivery in accordance with the wishes and needs of care recipients and informal caregivers. However, there is an apparent scarcity of research on the informal caregivers’ participation in the discharge planning. Current research underscores the importance of involving the informal caregivers early in the discharge process and encourages communication and information exchange between formal and informal caregivers. Research has identified a need to involve informal caregivers in the decision-making process to ensure successful post-discharge outcomes for the patient and the informal caregivers. However, we do not know enough about the specific roles of informal caregivers and their participation at and after the discharge process of older adults. This has become an issue of particular current interest due to proposed policy changes intending to develop a modern policy for informal care, more explicitly placing greater responsibility for a sustainable model of care on informal caregivers.

### Purpose

The purpose of this study is to describe the informal caregivers’ experiences of influencing decision-making at and after hospital discharge for home-bound older relatives. The specific research questions in this study were as follows: How do informal caregivers describe their role as participants in the decision-making concerning the health care services their older relative receives? How do informal caregivers describe their approach to influencing the care of their older relatives?

## Methods

### Setting and sample

**Figure 1:**
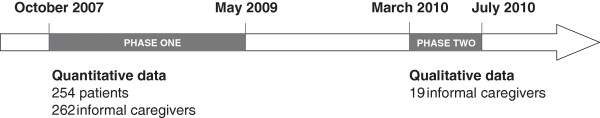
Timeline of data collection.

This exploratory, qualitative interview study is part of a larger research study that explored patients’ and informal caregivers’ participation in the discharge process during the transition from hospital to long-term primary health care in Norway. Recruitment of participants and data collection was carried out in two phases (Figure 1).

During Phase One, between October 2007 and May 2009, 254 patients and 262 informal caregivers from 52 municipalities were recruited to the study. Data were collected in structured self-report (face-to-face [patients] and telephone [informal caregivers]) interviews. The results from Phase One of the main study have been reported elsewhere [53,55,56].

**Figure 2:**
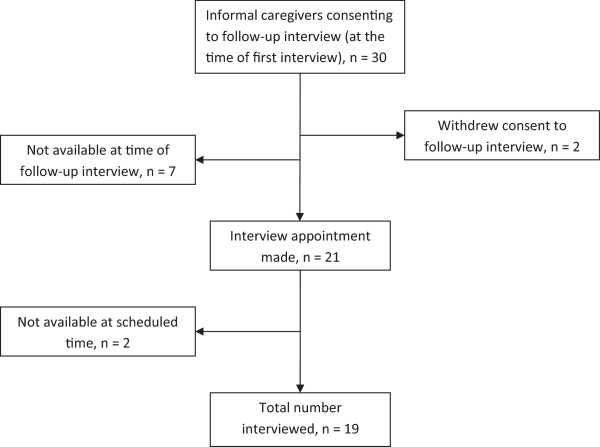
**Inclusion of informal caregivers during Phase Two.** Flow chart of inclusion of informal caregivers in the follow-up interviews.

During the last months of the data collection in Phase One, a sample of 30 informal caregivers of home-bound patients were asked for a preliminary consent to participate in follow-up interviews to be carried out at a later stage (Phase Two). The sample was chosen through a purposive sampling for maximum variation with the goal of selecting informal caregivers representing the range of experiences, kinship ties, and backgrounds [57]. During Phase Two, between March 2010 and July 2010, 19 informal caregivers gave their definitive consent to participate in the follow-up study (Figure 2). Qualitative telephone interviews were carried out with the 19 informal caregivers during Phase Two of the data collection.

### Interview guide preparation and data collection

A semi-structured interview guide was developed based on topics that emerged in the preceding structured interviews with informal caregivers [53]. In preparing the interview guide, audio recordings of a sample of 15 of the 262 previous interviews were utilized. The format of the structured interviews and the answers recorded in the questionnaires did not do justice to the stories of the informal caregivers; the audio recordings revealed their stories in greater detail. Thus, the research team decided to delve deeper into the experiences of the informal caregivers in the follow up study and encouraged the informal caregivers to express their experiences more freely in qualitative interviews. The three main themes of the interview guide were: (1) The role of the informal caregivers at and after discharge, (2) individual experiences of being an informal caregiver for an older relative, and (3) trust in the health care services.

At the beginning of the interviews, the informal caregivers were asked to talk about their experiences within the time frame from discharge up until the time of the follow-up interview. The initial question was: “Can you tell me what happened when your relative was discharged from the hospital?” This question allowed caregivers to start by telling their stories in their own words. Then, the interviewer continued by asking questions such as: “How would you describe your participation in the discharge process?”, “How would you describe your involvement with the formal caregivers in the municipality for follow-up care post discharge?”, “Did you experience any dilemmas as a caregiver in this process?”, and “In retrospect, what has been the most prominent experience?” The interview guide served as a reminder of the topics to cover and had suggested phrasings of questions but was not binding and did not structure the interviews in a uniform way. The purpose of the non-binding and semi-structured interview guide was to promote openness to follow the informal caregivers’ stories and explore their experiences. By choosing an open approach, we position the interviewer as an active participant in the construction of meaning in the interview [58,59]. The kinds of questions and follow-up prompts the interviewer used were influenced by her pre-understanding of the field of inquiry, consequently, the interviewer influenced the shared meaning production in the interview through her questions. The interviews lasted between 11 and 36 minutes with an average length of 24 minutes.

### Data analysis

All interviews were audio recorded, transcribed verbatim, and written out in their entirety in a normalized Norwegian language (not transcribing the informants’ dialect). Standardizing speech can make the informant’s meaning clearer; however, it can also eliminate elements that convey the distinctiveness and emotionality of the speaker [60]. We have strived to be faithful to what the person speaking wanted to convey; however, the transcription process is the first stage of interpretation, and this process is influenced by the researchers’ perception. The written representation of each interview has been filtered through our perception and our interpretation of the informant’s dialect and interpretation of what they wanted to convey. The text went through a second translation process from Norwegian to English for use in this article, again filtered through our perception and with our interpretation of the intended meaning and with our translation from Norwegian to English.

We selected a qualitative analysis inspired by an inductive thematic content analysis [57]. The initial stage of the qualitative analysis started with the transcription of the data material, and we completed this stage by reading through all transcripts and obtaining a general content overview of the material [57,61]. In the following description of our coding procedure and accounting for how categories and main themes were developed, we have strived to enhance transparency by accounting for the procedures we have used and the choices we have made.

### Coding procedure

We imported all interview transcripts into the qualitative analysis software HyperRESEARCH [62] and started the coding procedure. We developed codes inductively on the basis of the empirical data [57]. The HyperRESEARCH software program was used in this process of developing and keeping track of all the codes and coded passages of text from each of the interviews. To ensure a consistent coding practice in all 19 interviews, regardless of in what stage of the coding process the interview appeared, we read through the transcripts a second time when all the codes were created and added later codes where appropriate. As a conclusion of this step of the analysis we inspected all the codes in our codebook to determine if any of the codes overlapped and captured the same concepts and could be grouped together; we ended up with 52 unique codes in our codebook. This process concluded the code development, and the use of HyperRESEARCH software was discontinued at this stage of the analysis.

### Categorization and development of themes

Based on the codes, we grouped similar codes together in categories. We read the interview text, the codes, and categories several times in an iterative process through which we developed the main themes [57,61]. During these discussions, we reached a consensus about which codes and themes should be given priority in the subsequent analysis. At this point, the research question and the purpose of the study contributed to guiding our selection of codes and categories to prioritize. In the process of analyzing the interviews we emphasized an exploration of the categories and themes most prominently accentuated by our informants. Thus, some categories introduced by the researcher during the interview were not explored further because the empirical data did not support these categories as substantial concerns to our informants [57]. In the iterative process of analysis for this article, two main themes emerged in our interpretation of the empirical data material (Table 1). The first theme was “taking an active role.” The categories “emerging dependence” and “feelings of responsibility” were examples of the categories contained in this theme. Several codes were incorporated in these two categories, and two examples are presented in Table 1. The second main theme was “struggling to gain influence” (Table 1). In this main theme, categories such as “Working with the ‘gatekeepers’ of the health care services” and “strategies used when participating on behalf of the older relative” were included.

**Table 1:** Examples of codes, categories, and main themes of the qualitative analysis

**Transcribed text**	**Code**^**1**^	**Category**	**Main theme**
“My mother can’t pick up the phone to inquire about anything these days, so I’m the one who has to take over these tasks that she managed herself earlier. Because I am the only one capable of letting them [the municipality] know when something is not right.” (IC-10)	Being an informal caregiver involves looking after the older relative’s needs	Emerging dependence	Taking an active role
“It is important that I can act as a spokesperson, because she is not able to herself. [. . .] Being an intermediary sort of lies within the role, I think. It is part of the responsibility of [family members]” (IC-31)	Being an informal caregiver involves being the older relative’s spokesperson	Feelings of responsibility	
“It’s difficult for them [the home nurses] too, they may communicate our wishes, but their directives are not necessarily supported or acted upon. […] They understand our situation and are attentive towards us, but ultimately they don’t make the decisions.” (IC-10)	The decisions are not made by the home nursing providers	Working with the “gatekeepers” of the health care services	Struggling to gain influence
“After her breast surgery they wanted to send her home on a Friday. Her surgical wound was still open and it was . . . well, I outright declined. I said: ‘I am leaving town for the weekend, I will not be home if she is discharged’. . .” (IC-19)	You have to be resourceful to be heard	Strategies used when participating on behalf of the care recipient	

^**1**^The codes represent the quoted text in the context it appeared in the transcripts; the modified quotes used in this paper do not incorporate the full context that the code refers to.

### Ethical considerations and informed consent

This study was designed in accordance with the ethical principles for medical research involving human subjects as stated in the World Medical Association’s Declaration of Helsinki [63]. Approval for the study was obtained from the South-East Norway Regional Ethics Committee for Medical Research (reference number: 1.2007.1250) and all municipalities involved in the process of recruiting respondents. The study was reported to the Data Protection Official for Research (NSD) (project number: 17078). When the informal caregivers were approached for the follow-up interviews, all were informed about the status of the project and the progress since their initial interview. They were informed about the purpose of the follow-up interviews and assured that their data would be treated with confidentiality. During the process of transcribing the interviews, all names of municipalities, hospitals, and persons were removed and the informal caregivers were given anonymized identifying numbers that were used throughout the research process in all transcripts of the interviews and for the quotes used in this manuscript. They were informed about their right to withdraw their consent at any time for any reason. Lastly, they were asked to confirm their preliminary consent for participation and asked to consent for audio recording of the interview. All 19 informants gave their consent.

### Trustworthiness

To ensure the trustworthiness of the findings reported in this article, we focused on addressing a number of criteria determining the quality of qualitative research [57,58]. The research team’s experiences as health care personnel, informal caregivers to older family members, and as researchers conducting the preceding quantitative study have influenced our pre-understanding of the field of research. We have aimed for transparency in reporting our data analysis procedures [57], accounting for the use of HyperRESEARCH in our coding process and supplying examples of how the interviews were coded and categorized into main themes exemplified in a table showing examples of statements, codes, categories, and main themes (Table 1). We acknowledge that the data transcripts may have multiple readings. To maximize the legitimacy of our interpretations, all members of the research team took part in reading the transcripts, identifying the main themes, and discussing the emerging results until a consensus was reached on the interpretation of our findings [57,61]. The interpretations we present are influenced by the experiences of the research team and are inextricably linked to our perceptions as researchers. We assert that the collective effort to analyze the empirical material serves to counteract individual biases and strengthens the credibility of our interpretations. Furthermore, the quotes used in the article are intended to illustrate our interpretations of the informants’ statements and lend support to the trustworthiness of our analysis [60]. The use of quotes is also a way of introducing transparency to our analyses. We have attempted to account for the role of the researchers by reflexivity regarding our roles as co-creators of the data and the meaning presented in our results [57,58]. Altogether, these efforts were undertaken to ensure the trustworthiness of our findings and the conclusions made in this study.

## Results

### Participants

Thirteen women and six men were interviewed for this study. The informal caregivers included two spouses, thirteen sons/daughters, two daughter-in-laws and two nephews. At the time of the interview, participants were between 45 and 83 years of age with an average of 60 years. Eleven were gainfully employed in a part- or full-time position, and the remaining eight were retired or on disability benefits. During the time since our initial interview, some older relatives were admitted, sometimes more than once, to the hospital and discharged again, and six of them had passed away. Eight of the older relatives were now living independently in their private homes but were still receiving formal home health care services. Three of the relatives lived in sheltered housing provided by the municipality, and two had moved to a nursing home.

### Taking an active role

#### Emerging dependence and feelings of responsibility

The informal caregivers describe the older relative’s deteriorating health and declining self-care capacity as a starting point in their caregiver trajectory. One daughter explains that she was forced to take over tasks her mother previously managed due to her mother’s steadily declining function and increased frailty:

“My mother can’t pick up the phone to inquire about anything these days, so I’m the one who has to take over these tasks that she managed herself earlier. Because I am the only one capable of letting them [the municipality] know when something is not right”. (IC-10)

The informal caregivers convey that older relatives become dependent on help from their families:

“It is important that I can act as a spokesperson, because she is not able to herself. […] Being an intermediary sort of lies within the role, I think. It is part of the responsibility of [family members]” (IC-31)

These accounts highlight how the informal caregivers feel it’s necessary for them to take an active role to be able to influence the decision-making on behalf of their older relative. By taking on a role as spokesperson and intermediary they seek to ensure the needs of their older relative are heeded in the decision-making process.

A recurring feature of the informal caregivers’ descriptions is their extensive feelings of responsibility for the older relative’s well-being. Some convey that the feelings of responsibility are a natural part of what can be expected from family members, while other caregivers express the responsibility as a sense of duty toward their older relatives:

“Of course you feel the pressure, maybe not pressure exactly, but more that it is your duty to do the best you can. And that is part of your responsibilities, so to speak, as long as you have an old kin…” (IC-8)

In their adherence to filial obligation norms, where the ideal of reciprocity is a central tenet, the informal caregivers communicate their moral values, sense of duty, and emotional motives as strongly contributing to taking on the caregiving responsibility.

### The complexity of the health care services

The caregivers expressed their perception of the health care services as multi-faceted, hierarchical, and unpredictable and sometimes too complex to grasp. The informal caregivers view understanding the health care services as essential to taking an active part on behalf of their older relative. One daughter-in-law described herself as resourceful and knowledgeable about the organizational tiers of the health care services and usually capable of finding the right authority for her questions. She summarized her experiences:

“Me, I had, in a way, information about where to turn for help and sort of enquired in places where I could get more information and where I could turn for help and such. (…) It was very clear to me after a while that you have to be well informed as an informal caregiver to be able to make it through. You have to be quite resourceful. (…)” (IC-12)

Some informal caregivers found it difficult to participate in and influence care arrangement decisions because they did not know the services well enough:

“The challenge was all the things I didn’t know, things my wife could have received assistance with [from the municipality], but I didn’t know what to ask for (…)” (IC-19)

Despite apparent expectations to the contrary, some informal caregivers felt that it became their responsibility to monitor and assess the older relative’s health care needs and subsequently initiate contact with the health care services when formal care was needed:

“You can say that we felt that the informal caregiver sort of needs to be active. No one will seek you out to provide services. No one! Unfortunately, you have to take action yourself”. (IC-12)

This clearly shows that if the informal caregiver does not understand the services or know where to obtain assistance when the older relative’s health declines, the older individual and the informal caregiver are vulnerable. However, when the caregivers understand the services and have the resources to take an active role, the outlook is better:

“You know, you have to be very strong to make it, actually to be able to follow through with it. Yes, you have to! You can talk… and nothing happens, but we did it. […] They [the municipality] thought everything was fine. Until we put our foot down […]. It all worked out in the end”. (IC-23)

The older relative’s widespread dependency emerges through the informal caregivers’ descriptions. Through their accounts, it becomes clear that the health care services can be too complex if you are not able to be an active care recipient. The informal caregivers have to take the care recipient’s place and act as an intermediary between the relative and the health care services. In essence, the informal caregivers take the role of the active participant on behalf of their older relative.

### Struggling to gain influence

#### Working with the “gatekeepers” of the health care services

Many caregivers in our study expressed that they are at the mercy of individual health care personnel and case workers or contact persons. They described the health care personnel working in the purchaser unit of the municipality, and sometimes the personnel at the hospital, as “gatekeepers” guarding access to highly sought-after services. This widespread perception was expressed by several caregivers explaining how they felt they needed the goodwill of the case worker to participate in the process and that they were dependent on the case worker’s skills and willingness to advocate for the care recipient’s and caregiver’s wishes:

“Yes, absolutely, I feel that my opinions were heard [by our case worker]. She was a good person, she was very good at following up […] and I do think she did the best she could… But, of course she was no magician! She could only do so much”. (IC-12)

Statements like this further support the perception that caregivers and care recipients are at the mercy of the personnel in the health care services:

“It’s difficult for them [the home nurses] too. They may communicate our wishes, but their directives are not necessarily supported or acted upon. […] They understand our situation and are attentive toward us, but ultimately they don’t make the decisions”. (IC-10)

The informal caregivers were aware that the authority of the case workers was limited, acknowledging that the case workers were just a “cog in the machinery”:

“Yes, we had to fight. Because… well actually, I think the communication between the hospital and the municipality was greatly lacking. The hospital was clear on the fact that she had no business being discharged to her home in her condition, but at the nursing home they evaluated her situation differently and thought she was in excellent condition to manage at home with a bit of supervision”. (IC-12)

The informal caregivers try to make sense of the decisions that are made, which are not always predictable and can be the opposite of the agreements negotiated with the “gatekeepers”. The unpredictable outcome of decisions is reported as frustrating. However, the informal caregivers are careful to not be too openly critical of the services and the health care personnel working there because they are dependent upon the provided services; they do not wish to aggravate the service providers and risk losing the support. Several informal caregivers expressed this notion. One daughter explained that she had to restrain her critique toward the representatives from the municipality:

“Because, you know, I have to stay in their good graces because I am dependent on their help”. (IC-13)

Despite a widespread feeling of a personal responsibility for their spouses, elderly parents or extended family members, the informal caregivers expressed apprehension with being dependent on goodwill from the municipal health care services in their struggle to influence care decisions.

### Strategies used when participating on behalf of the older relative

All of the informal caregivers in our study took their responsibilities seriously. However, the informal caregivers chose different approaches to positioning themselves for gaining influence and they handled the ensuing challenges in different ways. One son describes what we have interpreted as a passive strategy of participation:

“I feel that it is important to participate, but I feel it is important to participate in a withdrawn way and rather contact the formal services if I discover that something is wrong or that they are neglecting to do certain things. I feel it is better to let them take the responsibility. Then, I can initiate dialogue if things are not working”. (IC-8)

This strategy is an example of the informal caregivers taking on a supervisory role, keeping tabs on the formal services, and reacting only when they uncover threats to what they consider to be the appropriate care for their older relative.

A daughter described a more active approach toward gaining influence. She and her husband fought a difficult battle with the municipality to have her mother placed in a nursing home following her hospitalization. The daughter describes an exhausting process of unsuccessfully advocating for her mother’s well-being during a period of frequent re-hospitalizations. Her attempts at establishing a dialogue with the municipality failed, and their applications for a nursing home placement were denied several times. The daughter finally resorted to stepping outside the chain-of-command in the municipality, contacting the administrative leader of the municipality directly:

“It all worked out in the end. But it is a pity that you have to go through all this before you are heard…It was terrible. I felt it was degrading that I had to fight with [the municipality]. I cried when I talked to those people, because I felt it was a terrible situation that we had to struggle with… all I wanted was for Mother to be properly cared for in her last years”. (IC-23)

When the informal caregivers reach a point where the situation is perceived as unbearable and all attempts at reaching agreements by dialogue fail, they resort to desperate strategies. One husband described how he resorted to making himself unavailable, knowing that the hospital could not safely discharge his wife if they knew she was on her own:

“After her breast surgery they wanted to send her home on a Friday. Her surgical wound was still open and it was… well, I outright declined. I said: “I am leaving town for the weekend, I will not be home if she is discharged”…” (IC-19)

A daughter used a similar strategy:

“I simply said “this will not work!” and I removed her keys and everything to prevent them from discharging her and sending her home in a taxi”. (IC-13)

These desperate actions are expressions of the informal caregivers’ struggle to gain influence and demonstrate that the care recipient’s safety is compromised without their cooperation. We found that some of the approaches toward participation and gaining influence were the result of exhausting all other options and resorting to measures that would force the services to acknowledge their strongly held opinions. Achieving the goal of the best possible care for the care recipient seem to depend on the ability of the informal caregivers to manage a complex reality, relentlessly and persistently navigating the health care services on behalf of their older relatives and having the resources to choose appropriate strategies for gaining influence over decisions.

## Discussion

### Taking an active role

The informal caregivers describe their older relatives’ deteriorating health and declining self-care capacity as a starting point in their caregiver trajectory. In combination with the complexities of health care services, the extensive frailty prevents older care recipients from taking an active role in handling their practical care arrangements in cooperation with formal care service providers. This is when the informal caregivers describe that they step up to actively participate on behalf of their older relative. These findings are consistent with findings from a Swedish study in which older relatives became dependent on their families for negotiating help arrangements [37]. Current research, corroborated by findings from this study, has shown that informal caregivers can contribute to a more favorable outcome for their older relative by taking care of and advocating for their rights and wishes in the discharge process [45]. By taking an active role as participants in decision-making the informal caregivers demonstrate their willingness to assume responsibility for their older relative.

Despite universal health care coverage in the Nordic countries, including public provision of long-term care, family members have historically played a central role in negotiating and providing care and has continued to provide the same care levels following the introduction of formal health care services [17]. The findings from this study shows that the informal caregivers currently shoulder substantial responsibilities and that they are willing and able to cooperate with the formal health care services to make sure their older relatives is adequately cared for. The comprehensiveness of the roles informal caregivers assume is virtually unlimited. The informal caregivers describe their roles as encompassing that of hands-on caregiver, spokesperson, intermediary, and advocate. Contemporary white papers more explicitly than before acknowledge that informal caregivers have important roles in supporting older relatives [3,10,11]. The intention to develop a modern policy of informal care including caregiver support services and respite care to enable informal caregivers to combine caregiving responsibilities with gainful employment and other responsibilities [3,10,11] may be a step toward formal recognition of the vital roles informal caregivers play.

### Struggling to gain influence

Consistent with other European studies, the caregivers in our study describe a constant struggle to gain influence [52,64] and to participate in the care decision-making process for their older relatives [26] despite the explicit expectation of their involvement. In our study, the informal caregivers express that this struggle intensifies when an older family member experiences greater functional decline and his or her care needs increase. According to the informal caregivers, some older individuals experience a rapid decline, increasing the need for 24-hour supervision and attention rather acutely. That kind of monitoring is only available through institutional care in a nursing home, and the family is no longer able to provide the needed amount of care. Ideally, the welfare state takes over the caregiving by providing formal services when the care needs of the care recipient reach this point [27]. However, the development of the municipal care sector in recent decades has challenged this perception [15,65]. Accordingly, our informants described substantial challenges to navigating the health care services to acquire the needed care for their older relative. The current policy of aging in place coupled with an aging population and retrenchment of institutional care in the community puts pressure on the municipal resources and on the informal caregiver resources. Informal caregivers describe desperately trying to negotiate and fight the system to obtain the next level of formal community care. Our results suggest, contrary to the claims that informal caregivers experience fewer burdens in the Nordic welfare state [31], that informal caregivers see their roles as demanding. As long as they struggle with gaining access to what they feel is appropriate help for their older relatives, it is unlikely that the formal rights to access services in the welfare state mitigates their feelings of responsibility. This is consistent with a report on user participation in the health and care sector that shows that care recipients and their informal caregivers may experience incongruity between their formal rights to participate and the actual participation in decision-making they experience in their local municipality [66]. In essence, the expectations of informal caregivers and care receivers are not always met with respect to their anticipated participation in decision-making, despite being formally stated in rules and legislation.

The informal caregivers in our study felt the need to resort to extreme measures to be heard by decision makers in the municipalities. They removed house keys or claimed to be leaving town to prevent their elderly relative from being discharged too early or to an empty house. They went outside the chain of command, appealing to the administrative leader of the municipality for their elderly relative to gain access to nursing home placement. These actions are desperate measures to force the decision makers or the gatekeepers to hear their arguments. In line with earlier research [52,53], our informants’ attempts at negotiations seemingly failed due to scarce opportunities for direct communication with the decision makers in the health care services. The only real chance of opposition is to claim serious deficits in patient safety, which is the only strategy that informal caregivers have found effective in communicating their disagreement with the care decisions. The informal caregivers express the responsibility they feel for the well-being of their older relative in a variety of ways and most prominently in the way they devote time and energy to making sure that their loved ones receive appropriate formal services. Informal caregivers do not always trust the formal health care services to take the appropriate responsibility [67], thus, the informal caregivers find themselves in a position of trying to mitigate the consequences of inadequate levels of care provided by the formal caregivers.

### Limitations of this study

This study is based on individual telephone interviews with a purposive sample of informal caregivers who have provided help and support to older relatives at and after discharge from somatic hospitals in Norway. The study is part of a larger study in which the research team have developed a questionnaire that patients and informal caregivers have answered through structured interviews. Based on past experiences and research in the preceding sub-studies, our assumption was that the role of informal caregiver would be important and complex, and that their experiences of participation would vary. These elements are parts of the authors’ pre-understanding of the field of research, which has in turn influenced the findings of this study. We encourage caution in generalizing the results from this study to other populations or other countries. The participants’ potential motivation for taking part in the study can be an important consideration when examining the trustworthiness of the results of the study. The informants in our study did not receive any material or economic incentives to participate, but some did express that they enjoyed the opportunity to share their thoughts and experiences with an interested party. Also, it is reasonable to assume that the informants have a subjective interest in the topic of the study, seeing as they within the last 12–18 months experienced their older relative’s discharge process. It is possible that informal caregivers with unique experiences were recruited. A unique story may have prompted the interviewer to ask for their participation in the follow-up interview, and the informal caregivers may have wanted to share their unique story, especially if they felt they contributed to a positive outcome for their older relative or if they faced difficulties and may have wanted to express their criticism of the system.

## Conclusions

Informal caregivers willingly take on the role as an intermediary between the care recipient and the health care services. This study shows that they take on the responsibility to seek information and establish dialogue with the formal health services in the municipality to negotiate sufficient formal services for their older relative. They recognize that their older relatives are unable to take the active participatory role that is needed, and in essence the informal caregivers actively participate on behalf of the care receiver and negotiate with the formal home health services to ensure that the best possible care is provided.

The informal caregivers describe how they exert a great deal of resourcefulness to be able to actively participate in and facilitate cooperation with health care services. The caregivers utilize different strategies and they identify establishing cooperation with the gatekeepers as a key strategy to be able to influence decision-making at and after discharge. The success of informal caregivers depends on several elements. First, informal caregivers must be willing to actively participate on behalf of their older relatives. Second, they have to devote relentless efforts and persistence to managing the complexities of the health care services. Last, they have to be able to choose appropriate strategies in order to gain influence. The care recipients’ extensive frailty and increasing dependence on their families coupled with the complexity of health care services contribute to the perception of the informal caregivers’ indispensable role as intermediaries.

### Implications

These findings accentuate the need to further discuss how frail older individuals and their informal caregivers can be supported and enabled to participate in decision-making regarding care arrangements that meet the care recipient’s needs. Failing to do so has the potential for becoming a serious deficit in our future care services, which is especially daunting when we recognize that informal caregivers are paramount in securing high-quality care arrangements for their older relative. The profound responsibility informal caregivers feel for the well-being of their older relative and how indispensable they appear to be when their older relative becomes dependent upon their support raises the question whether care recipients with strong, resourceful informal caregivers may receive qualitatively better care than recipients without caregivers or those with informal caregivers not strong enough to advocate and negotiate on their behalf?

## Competing interests

The authors declare that they have no competing interests.

## Authors’ contributions

LKB, MK and CF participated in the study conception and design. LKB performed the majority of the data acquisition, analysis and interpretation in addition to drafting the manuscript. MK and CF contributed to the analysis, interpretation of the data and manuscript revisions. All authors read and approved the final manuscript.

## Pre-publication history

The pre-publication history for this paper can be accessed here:

http://www.biomedcentral.com/1472-6963/14/331/prepub
